# The feasibility of paramedics delivering antibiotic treatment pre-hospital to ‘red flag’ sepsis patients: a service evaluation

**DOI:** 10.29045/14784726.2018.03.2.4.19

**Published:** 2018-03-01

**Authors:** Jonathan Chippendale, Adele Lloyd, Tanya Payne, Sally Dunmore, Bethan Stoddart

**Affiliations:** East Midlands Ambulance Service NHS Trust; North Lincolnshire and Goole NHS Trust; East Midlands Ambulance Service NHS Trust; East Midlands Ambulance Service NHS Trust; Path Links

**Keywords:** ambulance, bacteraemia, paramedic, pre-hospital care, sepsis, sepsis 6, septic shock

## Abstract

**Background:**

Sepsis is associated with a 36% mortality rate, rising to 50% for septic shock. Currently, when an East Midlands Ambulance Service clinician recognises ‘red flag’ sepsis, only the oxygen and fluid elements of the ‘Sepsis Six’ care bundle are delivered, omitting the antibiotic therapy. For a patient in septic shock, every hour’s delay in antibiotic therapy is associated with a 7.6% increase in mortality. Ambulance clinicians are therefore appropriately placed to assess and commence treatment at the earliest point of recognition. The aim of this evaluation was to assess the feasibility of training paramedics to recognise ‘red flag’ sepsis, obtain blood cultures and administer a broad spectrum antibiotic, meropenem, to patients in the pre-hospital environment.

**Methods:**

A prospective six-month feasibility pilot evaluation was conducted in May 2016. Paramedics were trained and given access to a broad spectrum antibiotic, meropenem, along with a patient group direction to administer the antibiotic to ‘red flag’ sepsis patients. Training included sepsis recognition, taking of blood cultures and patient group direction compliance.

**Results:**

Twenty paramedics volunteered and successfully completed the training. Of the 113 patients that were identified as ‘red flag’ sepsis, 107 (94.6%) were confirmed as infected by the receiving hospital. Ninety-eight blood samples were successfully drawn by study paramedics, with only seven (7.1%) reported as contaminated samples, compared with 8.5% of samples taken by staff in the receiving ED during the same time period. Ninety patients (80%) assessed by paramedics as meeting the criteria were treated with meropenem, and patient group direction compliance was 100%.

**Conclusion:**

Paramedics can safely deliver pre-hospital antibiotics to patients with ‘red flag’ sepsis and obtain blood cultures prior to administration, with a contamination rate comparable with local hospitals, following a short training course.

ED Procedure – Forms summary for patient receiving Meropenem
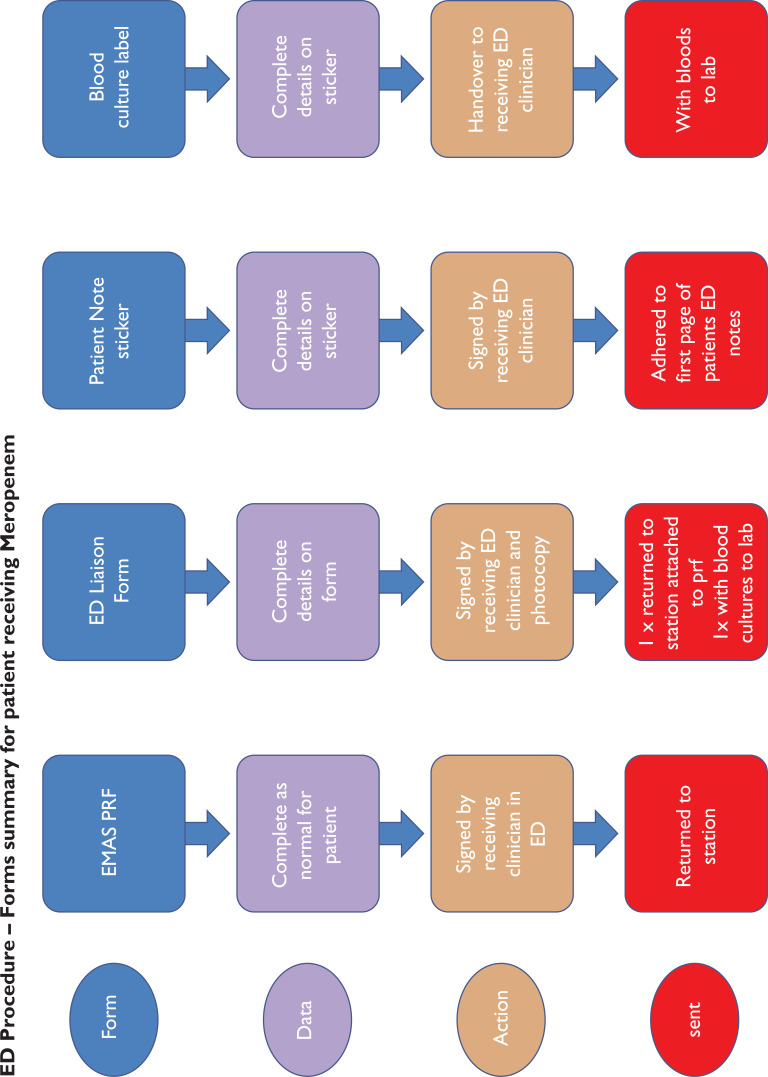

ED Procedure – Forms summary for patient NOT receiving Meropenem
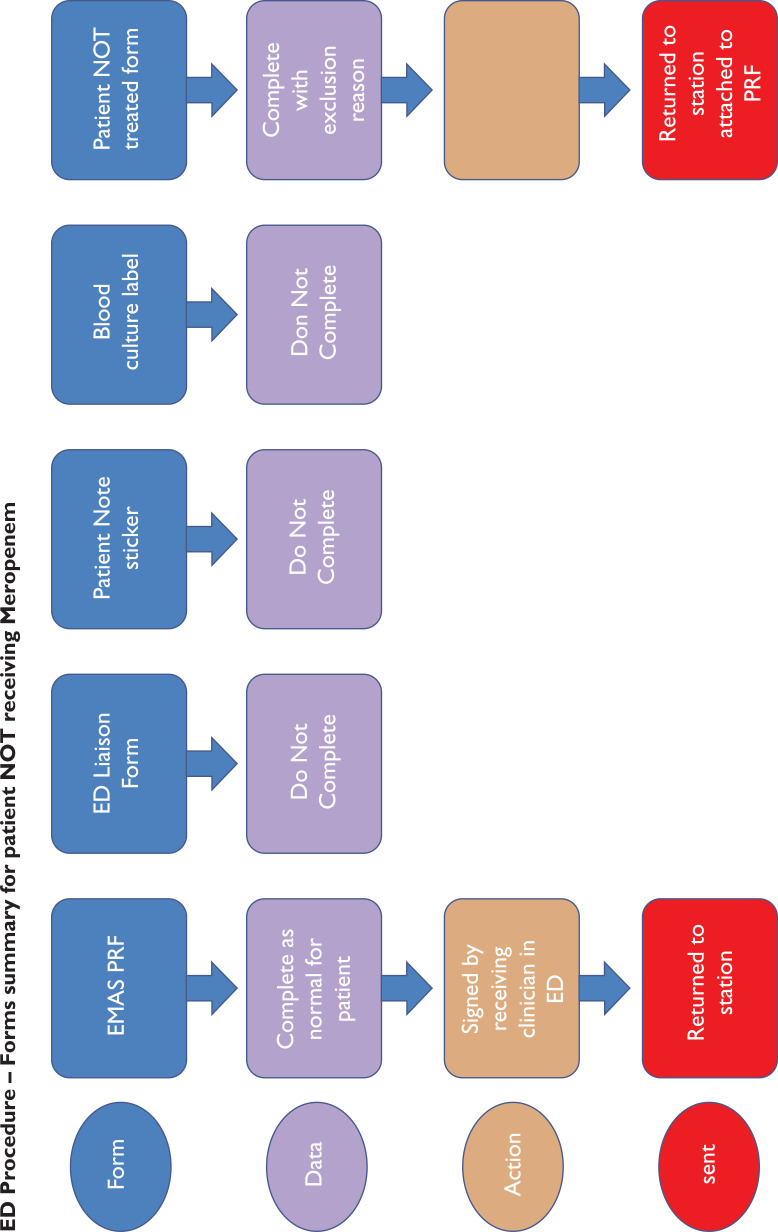

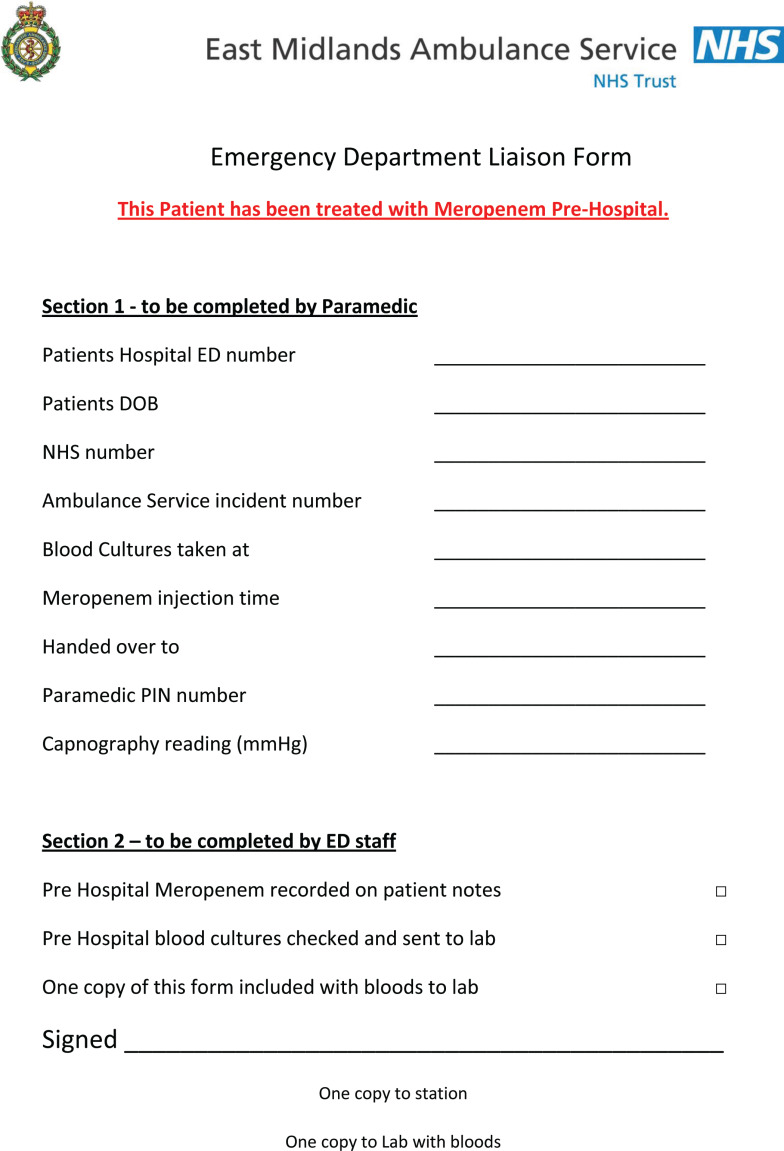



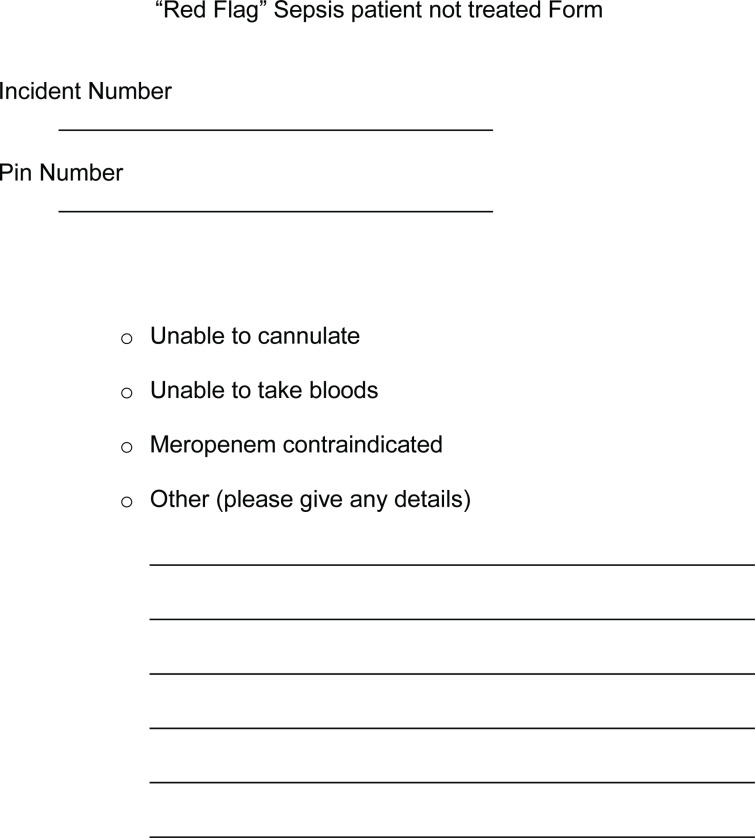

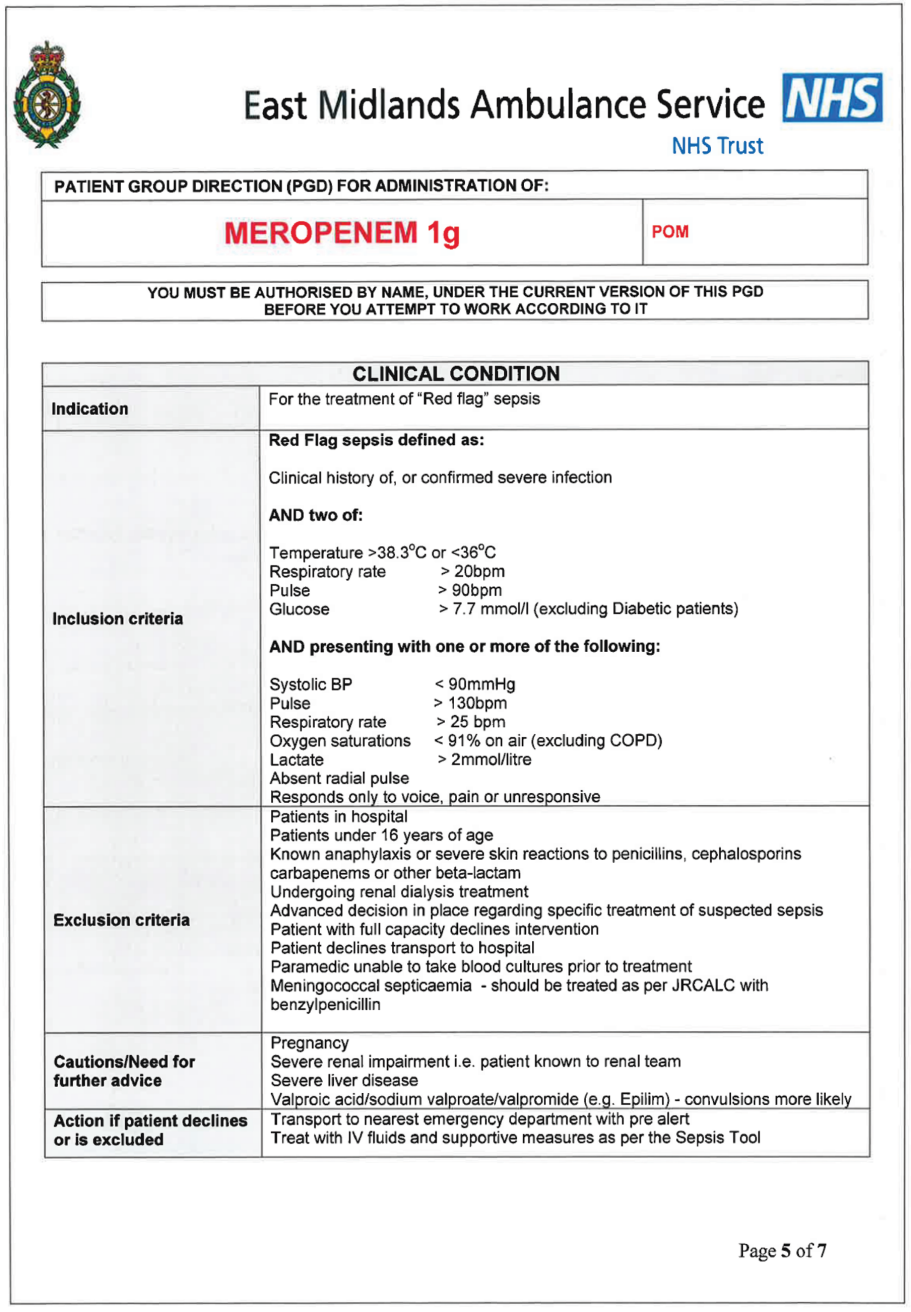



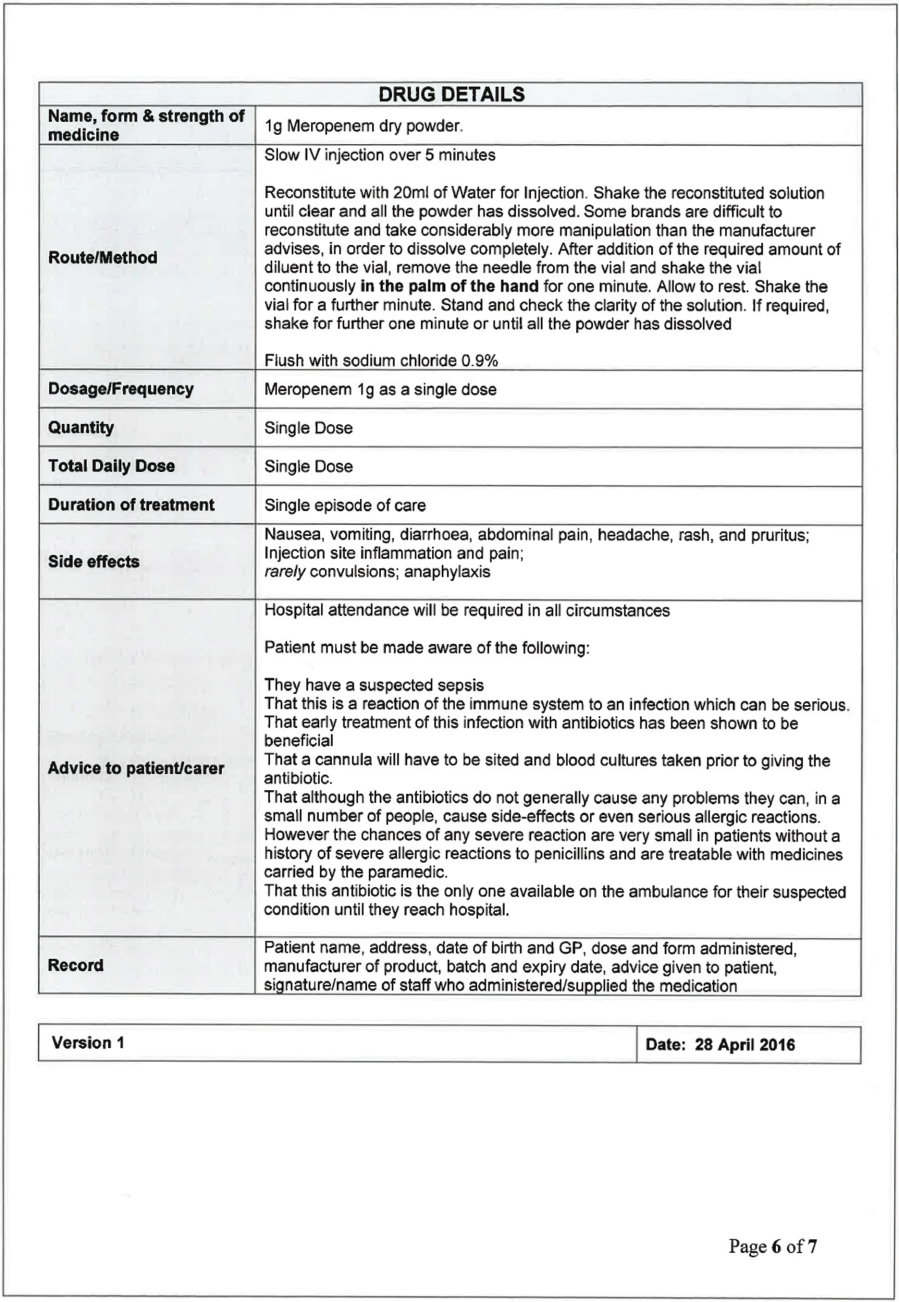

## Introduction

East Midlands Ambulance Service NHS Trust (EMAS) provides emergency 999 care across the five counties of Derbyshire, Leicestershire, Lincolnshire, Northamptonshire and Nottinghamshire, serving a population of 4.8 million people. Through the year from 2015 to 2016, EMAS received 651,000 calls.

The UK Sepsis Trust (2015) defines sepsis as a time-critical condition that can lead to organ damage, multi-organ failure, septic shock and eventually death. It is caused by the body’s immune response to a bacterial, fungal or viral infection. It commonly originates from the lungs, bowel, skin, soft tissues and urinary tract. Rarer sources include the lining of the brain (meningitis), liver or indwelling devices such as catheters. Sepsis causes changes in the circulation, reducing the blood supply to major organs such as the kidneys, liver, lungs and brain. Although most dangerous in those with impaired immune systems, it can be a cause of death in young and otherwise healthy people.

Historically, sepsis has been categorised through diagnostic criteria including a documented or suspected infection in addition to the presence of abnormal physiological variables. In addition, severe sepsis has been defined as sepsis plus organ dysfunction and septic shock as sepsis plus hypotension or hyperlactaemia (Levy et al., 2003).

Severe sepsis is responsible for at least 37,000 patient deaths and 100,000 hospital admissions in the UK per year (Daniels, 2011). Sepsis is associated with a 36% mortality rate (Vincent et al., 2006), rising to 50% in patients with signs of septic shock (Angus et al., 2001). Over 70% of sepsis cases arise in the community (Esteban et al., 2007).

Each hour’s delay of administration of antibiotics to patients with septic shock is associated with a 7.6% greater risk of death (Kumar et al., 2006). It is also recognised that there is a linear increase in mortality for each hour’s delay of antibiotic administration for patients with severe sepsis and septic shock (Ferrer et al., 2014). Considering that ambulance service clinicians may be the first healthcare professionals to assess the patient, paramedics are appropriately placed to make an early diagnosis and commence treatment.

It might seem sensible to advocate antibiotics for every-one if the risk of delay is so high; however, antibiotic stewardship makes this strategy unwise. Antimicrobial resistance (AMR) has been observed ever since the introduction of antibiotics and the indiscriminate and inappropriate use of antibiotics is a driver for the spread of AMR. In order to preserve the effectiveness of the anti-microbial treatments available, organisations need to ensure suitable antibiotic stewardship to stem overuse of antibiotics and slow the development of further AMR (Department of Health, 2013).

The UK Sepsis Trust has developed a screening tool to aid the rapid identification of ‘red flag’ sepsis, a subgroup of patients who have specific observations that can be rapidly measured or determined, and once identified, recommend delivery of the ‘Sepsis Six’, a set of six interventions: oxygen, blood cultures, antibiotics, fluids, lactate measurement and urine output monitoring (NHS England, 2014). They further suggest that treatment should be initiated within one hour of recognition (UK Sepsis Trust, 2015). At present, when an EMAS clinician recognises ‘red flag’ sepsis, only the oxygen and fluids element of the ‘Sepsis Six’ care bundle is delivered.

This service evaluation aims to explore the feasibility of a paramedic delivering an antibiotic, meropenem, to ‘red flag’ sepsis patients in the pre-hospital environment. However, since Public Health England (2014) recommend that blood culture samples should be collected before antimicrobial therapy where possible, the feasibility of paramedics taking blood culture samples prior to the administration of antibiotics was also evaluated.

## Methods

### Aim

The aim of this evaluation was to explore the feasibility of delivering antibiotic therapy to patients identified with ‘red flag’ sepsis in the pre-hospital environment.

### Outcomes

- Determine whether paramedics can appropriately deliver an antibiotic to ‘red flag’ sepsis patients.
- Calculate the blood culture contamination rate when blood is drawn in the pre-hospital environment by paramedics.

### Participants

The study was a joint collaboration between EMAS and North Lincolnshire and Goole NHS Hospital Trust (NLAG). Twenty EMAS paramedics from ambulance stations servicing the North Lincolnshire region volunteered to participate in the study. Recruitment was voluntary and participants self-volunteered by responding to locally placed adverts.

The training of participants was delivered by both Trusts in two phases. The initial training programme consisted of the sepsis recognition training as delivered to all EMAS clinicians during the financial year 2014 to 2015 as part of an ongoing EMAS essential education programme. In addition, a patient group direction (PGD) was developed so that study paramedics could administer the antibiotic, meropenem (Supplementary 1). The choice of antibiotic and subsequent dosage was made between senior representatives from EMAS and NLAG which included the NLAG Path Links Consultant Microbiologist, the NLAG Consultant Pharmacist and the Pharmacy Advisor from EMAS. Meropenem 1 g was the preferred choice of antimicrobial agent due to its effectiveness over a broad spectrum of bacteria, in addition to there being comparatively low incidences of adverse reaction in patients (Electronic Medicines Compendium, 2016). The pharmaceutical form of meropenem being reconstituted from a dry powder, and a dose of 1 g being delivered by intravenous injection, offered a consistent method of delivery in line with other medicines administered by paramedics within their existing scope of practice.

The second phase of the training took place within the emergency department at one of the NLAG hospitals and included instruction in the collection of blood culture samples. To alleviate the need to introduce new equipment for this evaluation, paramedics obtained blood cultures via a cannula.

### Procedure

On successful completion of the study training, the paramedics were each issued their own personal use sepsis bag which included the study materials and documents required for one patient (Supplementary 2). Meropenem was signed out from the drug cupboard on station at the start of shift and signed back in if not used.

Recruitment of patients was undertaken between March and October 2016. Patients were eligible for inclusion if they met the criteria based on the NHS England and UK Sepsis Trust safety bulletin from September 2014 (Figure 1).

**Figure:**
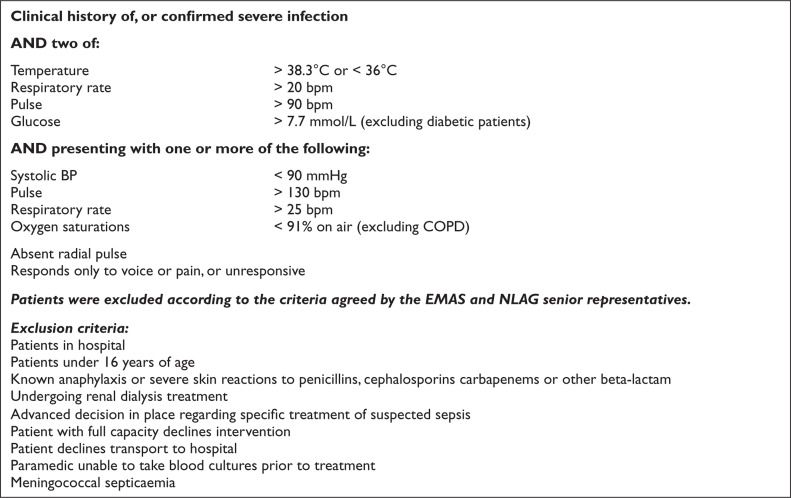
Figure 1. Inclusion criteria.

Full assessment and observations of each patient as per normal EMAS procedures were expected to be followed. Where ‘red flag’ sepsis was identified by a study paramedic, and the nearest receiving emergency department (ED) was Scunthorpe General Hospital (SGH) or Diana Princess of Wales Hospital (DPOW), Grimsby (i.e. NLAG hospitals), blood cultures were taken and meropenem administered, in accordance with the PGD. Where the patient was contra-indicated for treatment with meropenem, care was delivered as per normal EMAS, Joint Royal Colleges Ambulance Liaison Committee (2016) guidelines and transported to the ED.

### Data collection

In addition to the patient report form (PRF), study paramedics also completed a ‘patient treated’ or ‘patient not treated’ form for those patients recognised as ‘red flag’ sepsis. Both forms were processed using EMAS policy and procedures where they were identified by the clinical audit team and collected. The following study data were extracted and anonymised prior to analysis:

- 999 call date and time.
- Patient’s clinical observations including respiratory rate, pulse rate, blood glucose measurement, oxygen saturations, blood pressure, level of response, Glasgow Coma Scale and temperature.
- Time of blood culture harvesting.
- Time of antibiotic administration.

Subsequently, the following data were obtained from the receiving ED:

- Blood culture analysis report.
- Hospital pharmacy record.
- Hospital discharge summary.

All data were stored and managed on an EMAS NHS encrypted laptop computer with a back up stored on an EMAS NHS encrypted server.

### Safety

Monthly review sessions between the EMAS study lead and the NLAG sepsis nurse specialist confirmed which of the patients enrolled matched the definition of ‘red flag’ sepsis and which, by hospital record, were classed as infected. This was based on clinical opinion and supported by C-reactive protein (CRP) levels, white blood cell count (WBC), continuation of antibiotic treatment in hospital and the diagnosis recorded on the patient’s discharge summary. Blood cultures taken were tested for likely contaminants by NLAG microbiology as per normal procedures. Procedures were put in place to provide feedback and support to study paramedics who made an incorrect diagnosis and/or submitted contaminated blood culture samples.

## Results

Between March and October 2016, study paramedics identified 113 patients as ‘red flag’ sepsis. All patients met the ‘red flag’ sepsis criteria, 98 (86.7%) had blood culture samples drawn and 90 received meropenem. Compliance with the PGD was 100% (Figure 2).

**Figure:**
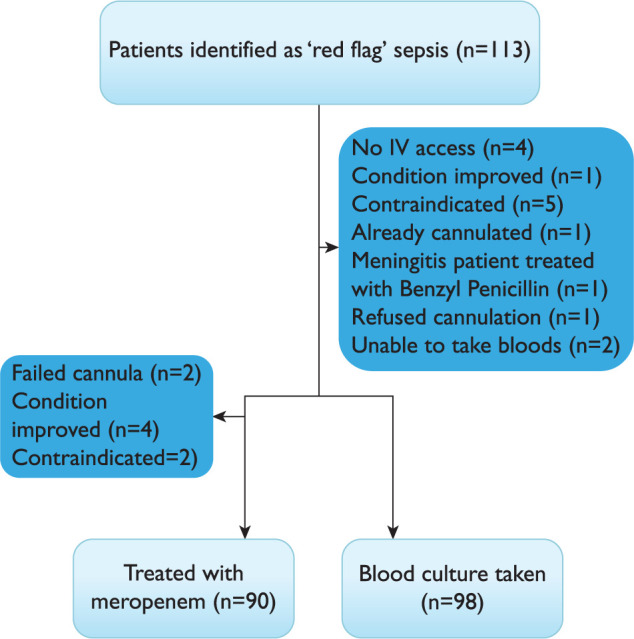
Figure 2. Patient flow diagram.

Following a review of hospital records, 107 (94.6%) patients included in the study were found to have an infection, one hospital record had no diagnosis and the remainder had a non-infectious cause (Table 1).

**Table 1.** Final diagnosis of patients falsely identified as infected.

Discharge summary diagnosis	Number of patients not found to have an infection
Pulmonary embolism (PE)	1
Transient ischaemic attack (TIA)	1
Cerebrovascular vascular accident (CVA)	1
Tumour (bladder)	1
No diagnosis recorded but not treated for infectious cause	1
Intra-cardiac thrombus attached to pacing wire	1

Fourteen paramedics out of the 20 taking part drew blood cultures during the study from 98 (87%) of the patient participants. Of the patients where a blood culture was harvested by a study trained paramedic, seven (7.1%) returned a result marked as ‘likely environmental contaminant’ from the investigating laboratory. This compared to contamination rates from the two receiving EDs of 7.9% and 9.1% over the same time period (Table 2).

**Table 2.** Blood contamination rates.

Source	EMAS study trained paramedic	ED (SGH)	ED (DPOW)
Contamination rate (%)	7.1	7.9	9.1

Four paramedics in total returned contaminated samples. One paramedic returned three contaminated samples, with a further paramedic returning two, and two paramedics returning one contaminated sample each.

## Discussion

With the limited point of care blood testing available in the pre-hospital environment, the UK Sepsis Trust (2015) refers to ‘red flag’ sepsis patients as being identified with a high index of suspicion, as opposed to a definitive diagnosis. While clinical observations are objective and mostly unequivocal, the criteria surrounding ‘clinical history suggestive of a severe infection’ is much more difficult to assess due to its subjectivity. However, in this study, only 6/113 (5.3%) patients ultimately were false positives, having been subsequently found not to have an infection (Table 1).

While the patients with the pulmonary embolism and bladder tumour were found to have histories consistent with that of a chest infection and urinary tract infection respectively, for three of the patients, a source of infection could not be determined. The patient with no eventual diagnosis recorded was suspected by the paramedic to have cellulitis. Although this condition was present, the hospital records showed that the patient received no further treatment for the infection and the patient’s symptoms resolved.

Closer review of the EMAS PRF against these patients highlighted that the study paramedic’s clinical history taking and documentation was suggestive of an infection, which was further supported by clinical observations matching the inclusion criteria for the PGD.

Four paramedics out of the 20 returned contaminated samples throughout the study. Two of them returned two contaminated samples each and went on to undertake a reflective session with the study lead to review their training and blood sample harvesting technique. After the review session, one continued to take blood throughout the remainder of the study without issue, whereas the other took contaminant free samples until the last week of the study before returning a further spoiled sample. This would have resulted in further training in accordance to the protocol, but the study period ceased before this could take place. However, this was still lower than that of the receiving EDs, but given that contamination rates are considered to be higher in blood cultures taken from a cannula as opposed to venepuncture (Self et al., 2012), continuous auditing of these rates is important.

### Limitations

The study represented a small number of clinicians over a limited six-month time span. This was reflected in the low number of patients recruited into the study. Additionally, those clinicians participating did so on a voluntary basis as opposed to being randomly selected, and attended a training session which included sepsis recognition as one of the learner outcomes. Therefore the study paramedic group was proactive and forthcoming, which may not accurately represent the wider paramedic population.

Study paramedics received refresher training in recognition of ‘red flag’ sepsis. This training was additional to that of the wider paramedic workforce and therefore may positively impact on the study outcome. However, no attempt was made during this evaluation to determine the number of false negatives – that is, patients with sepsis who did not receive antibiotics when they should have.

Finally, study patients were from a localised geo-graphical area and therefore may not be demographically representative of the wider population.

## Conclusion

Paramedics can safely deliver pre-hospital antibiotics to patients with ‘red flag’ sepsis and obtain blood cultures prior to administration, with a contamination rate comparable with local hospitals, following a short training course.

## Author contributions

JC: Overall management of the project and management within East Midlands Ambulance Service.

AL: Management of the project within North Lincolnshire and Goole NHS Hospital Trust.

TP: Research governance and procedures along with editorial contribution.

SD: Staff training, logistics and data collection.

BS: Management of the project within Path Links microbiology services and governance over antibiotic choice.

## Conflict of interest

None declared.

## Ethics

This study was classed as a service evaluation and so did not require HRA approval. Permission to conduct the evaluation was provided by East Midlands Ambulance Service NHS Trust Clinical Governance Group.

## Funding

None.
